# Assessment of brain reference genes for RT-qPCR studies in neurodegenerative diseases

**DOI:** 10.1038/srep37116

**Published:** 2016-11-17

**Authors:** Rasmus Rydbirk, Jonas Folke, Kristian Winge, Susana Aznar, Bente Pakkenberg, Tomasz Brudek

**Affiliations:** 1 Research Laboratory for Stereology and Neuroscience, Bispebjerg-Frederiksberg Hospital, University Hospital of Copenhagen, Bispebjerg Bakke 23, DK-2400, Copenhagen, Denmark; 2 Bispebjerg Movement Disorder Biobank, Bispebjerg-Frederiksberg Hospital, University Hospital of Copenhagen, Bispebjerg Bakke 23, DK-2400, Copenhagen, Denmark; 3 Department of Neurology, Bispebjerg-Frederiksberg Hospital, University Hospital of Copenhagen, Bispebjerg Bakke 23, DK-2400, Copenhagen, Denmark; 4 Institute of Clinical Medicine, Faculty of Health, University of Copenhagen, Blegdamsvej 3B, DK-2200, Copenhagen, Denmark

## Abstract

Evaluation of gene expression levels by reverse transcription quantitative real-time PCR (RT-qPCR) has for many years been the favourite approach for discovering disease-associated alterations. Normalization of results to stably expressed reference genes (RGs) is pivotal to obtain reliable results. This is especially important in relation to neurodegenerative diseases where disease-related structural changes may affect the most commonly used RGs. We analysed 15 candidate RGs in 98 brain samples from two brain regions from Alzheimer’s disease (AD), Parkinson’s disease (PD), Multiple System Atrophy, and Progressive Supranuclear Palsy patients. Using RefFinder, a web-based tool for evaluating RG stability, we identified the most stable RGs to be *UBE2D2, CYC1*, and *RPL13* which we recommend for future RT-qPCR studies on human brain tissue from these patients. None of the investigated genes were affected by experimental variables such as RIN, PMI, or age. Findings were further validated by expression analyses of a target gene *GSK3B*, known to be affected by AD and PD. We obtained high variations in *GSK3B* levels when contrasting the results using different sets of common RG underlining the importance of a priori validation of RGs for RT-qPCR studies.

Reverse transcription quantitative real-time polymerase chain reaction (RT-qPCR) is one of the most widely used methods for analysis of gene expression levels due to its methodological accessibility and high sensitivity. Studies on neurodegenerative diseases are no exceptions, and RT-qPCR have helped to identify gene expression changes and thereby disease-associated genes in several disorders, e.g. Alzheimer’s disease (AD)^1^,^2^,^3^,^4^, and Parkinson’s disease (PD)^5^,^6^,^7^. Identification of small, dynamic changes in gene expression is important for understanding of the mechanisms underlying neurodegenerative events. An essential step in the development of RT-qPCR assays is identification of appropriate reference genes (RGs) to be used as endogenous controls in data normalization. Housekeeping genes (HKGs) are often the most obvious choice as constitutive RGs since HKGs are important for basal cellular functions and should therefore exhibit stable levels under normal and pathophysiological conditions. However, it has been shown that expression levels of many HKGs can also be affected by disease-mediated pathological changes in, e.g., cell homeostasis and metabolism^8^,^9^. Thus, the selection of stable RGs is critical in order to avoid methodological errors. For many years, glyceraldehyde 3-phospate dehydrogenase (*GAPDH*) and beta(β)-actin (*ACTB*) have been the primary choices as RGs. Though, in the last decade a vast number of studies have documented that these genes should be used with caution since they may display variable expression levels across various cell types and disease states^10^,^11^,^12^,^13^,^14^.

Descriptive statistics is the most straightforward way for comparing RG expression stability. Even though widely applied, this approach has its pitfalls due to its simplicity. There are several more advanced methods available that address the problem of RG transcription stability taking into account multiple parameters. The four most common are the ΔCt-method^15^ and the three algorithms Genorm^16^, BestKeeper^17^, and NormFinder^18^ (reviewed elsewhere^19^). RefFinder, a free online software platform for identification of stable RGs, derives a comprehensive ranking based on the geometric mean of the scores from Genorm, BestKeeper, NormFinder, and the ΔCt-method in order to extract the best RGs based on the different mathematical approaches^20^. Although these methods differ in their mathematical design, studies have shown only small variations in the outcomes for Genorm, NormFinder, and BestKeeper, and also between the stand-alone applications for Genorm, NormFinder, and BestKeeper, and RefFinder, making these methods comparable^21^,^22^.

The overall aim for this study was to evaluate putative RGs for use in gene expression studies on the human brains in diverse, but pathologically related neurodegenerative diseases. Here, we report on 15 candidate RGs that potentially can be used for normalization of gene expression data in brain samples from patients diagnosed with AD, PD, Multiple System Atrophy (MSA), or Progressive Supranuclear Palsy (PSP) compared with normal, non-neurological affected controls (NCs). We selected RG candidates based on their extensive use in RT-qPCR studies from the literature as well as their presence in ready to use commercial kits for RG selection. Several of the RG candidates have previously been investigated under different conditions^10^,^12^,^23^,^24^,^25^,^26^, and a few have been tested in AD and/or PD^11^,^27^,^28^. MSA and PSP are atypical parkinsonian disorders and have a clinical manifestation highly similar to PD^29^. To our knowledge, no study on RG selection has been performed on MSA or PSP thus far. Hence, assessment of stable RGs for comparative studies on PD, MSA, and PSP is highly relevant in order to strengthen the reliability of the experimental outcome. Furthermore, although PSP is clinically comparable to PD, the pathological events in PSP are similar to AD^30^. We investigated two disease-affected brain regions, the medial frontal gyrus of the prefrontal cortex (PFC), and the cerebellum (CB), in order to define the most stable set of RGs for comparative gene expression studies in PD, MSA, PSP, and AD brains. The following genes were investigated (Table 1): *GAPDH*, *ACTB*, ribosomal protein large 13 (*RPL13*), hypoxanhine phosphoribosyl transferase (*HPRT1*), cytrochrome C1 (*CYC1*), topoisomerase 1 (*TOP1*), eukaryotic translation initiation factor 4A2 (*EIF4A2*), β-2-microglobulin (*B2M*), pumilio-homolog 1 (*PUM1*), TATA-box binding protein (*TBP*), ubiquitin C (*UBC*), cyclophilin A (*PPIA*), succinate dehydrogenase complex subunit A (*SDHA*), ATP synthase H+ transporting mitochondrial F1 complex beta polypeptide (*ATP5B*), and ubiquitin-conjugating enzyme E2D2 (*UBE2D2*). Further, glycogen synthase kinase 3β (*GSK3B*) was included as a Gene of Interest (GOI) to validate the impact of the RG selection on normalization of gene expression data.

## Results

### RT-qPCR primer amplification efficiencies

Primer efficiencies were calculated for each candidate RG and ranged between 90.4% and 107.6% using a minimum of four points on the standard curve with *R*^*2*^-values ranging from 0.992 to 1.000 (Table 1). Each reaction ended with a melting curve analysis between 55 and 95 °C for PCR product evaluation (Fig. S1). If melting curve analysis identified presence of primer dimers, an additional acquisition step was added in each PCR cycle (Table 1) since primer dimers do not generate signal in fluorescence captured over 76 °C. Due to low expression levels in both brain regions (cycle threshold (Ct)_mean_ > 35) and since it was not possible to obtain good efficiency for *HPRT1* and *SDHA,* the genes were not included in the subsequent stability measurements.

### Descriptive statistics of candidate RGs

**Figure 1:**
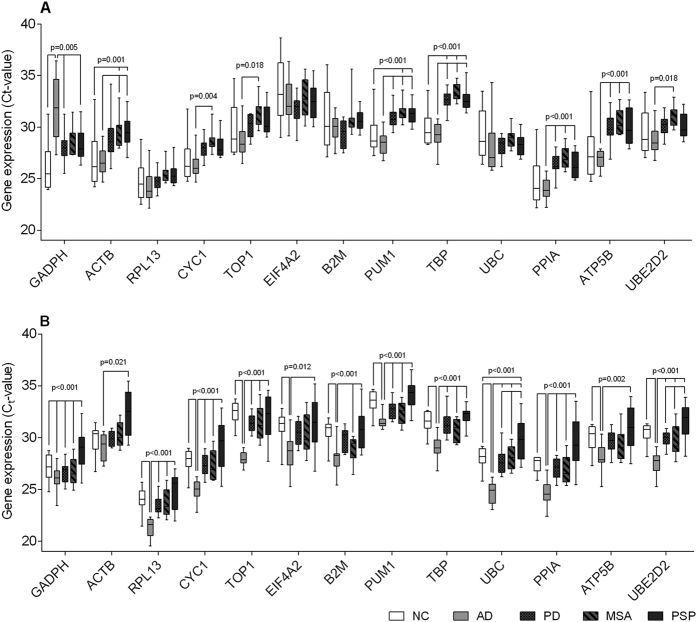
Thirteen candidate reference gene Ct-value distributions in both brain areas. Box and whisker plots showing RT-qPCR mRNA expression levels depicted as raw Ct-values in the prefrontal cortex (**A**) and the cerebellum (**B**) for all Alzheimer’s disease (AD), Parkinson’s disease (PD), Multiple System Atrophy (MSA), Progressive Supranuclear Palsy (PSP), and normal, non-demented controls (NCs). Boxes depict the 25^th^, 50^th^ and 75^th^ percentiles; whiskers show the minimum and maximum values for each RG. *p*-values indicate the ANOVA significance levels.

Almost all candidate RGs showed significantly aberrant expression levels between the disease groups in both brain regions with the exception of *RPL13, EIF4A2, B2M,* and *UBC* in the PFC which showed no significant differences between groups (Fig. 1). Ct-values for the 13 included candidate RGs in the PFC and CB for all five groups ranged between 19.9 and 38.7 (Fig. 1, Table 2). The standard deviations (SDs) ranged between 0.66 and 3.09 (Table 2). In the PFC, *EIF4A2* had the lowest expression levels (mean Ct (Ct_mean_) = 32.6) followed by *TBP* (Ct_mean_ = 31.5), and *B2M* (Ct_mean_ = 30.3). In the CB *PUM1* (Ct_mean_ = 32.8), *TOP1* (Ct_mean_ = 31.0), and *TBP* (Ct_mean_ = 30.9) exhibited the lowest expression levels. The genes with the highest expression levels were in both brain regions *RPL13* (Ct_mean, PFC_ = 25.0 and Ct_mean, CB_ = 23.5), and *PPIA* (Ct_mean, PFC_ = 25.7 and Ct_mean, CB_ = 27.1). In all groups, *EIF4A2* showed the most variable expression levels in both regions reflected by the mean SD (SD_mean, PFC_ = 2.18 and SD_mean, CB_ = 1.80). In both brain regions, the RG candidates that exhibited the lowest variability in expression levels were *TBP* (SD_mean, PFC_ = 1.21 and SD_mean, CB_ = 1.09), *PUM1* (SD_mean, PFC_ = 1.24 and SD_mean, CB_ = 1.05), and *RPL13* (SD_mean, PFC_ = 1.33 and SD_mean, CB_ = 1.24).

### Summarized comprehensive ranking

**Figure 2:**
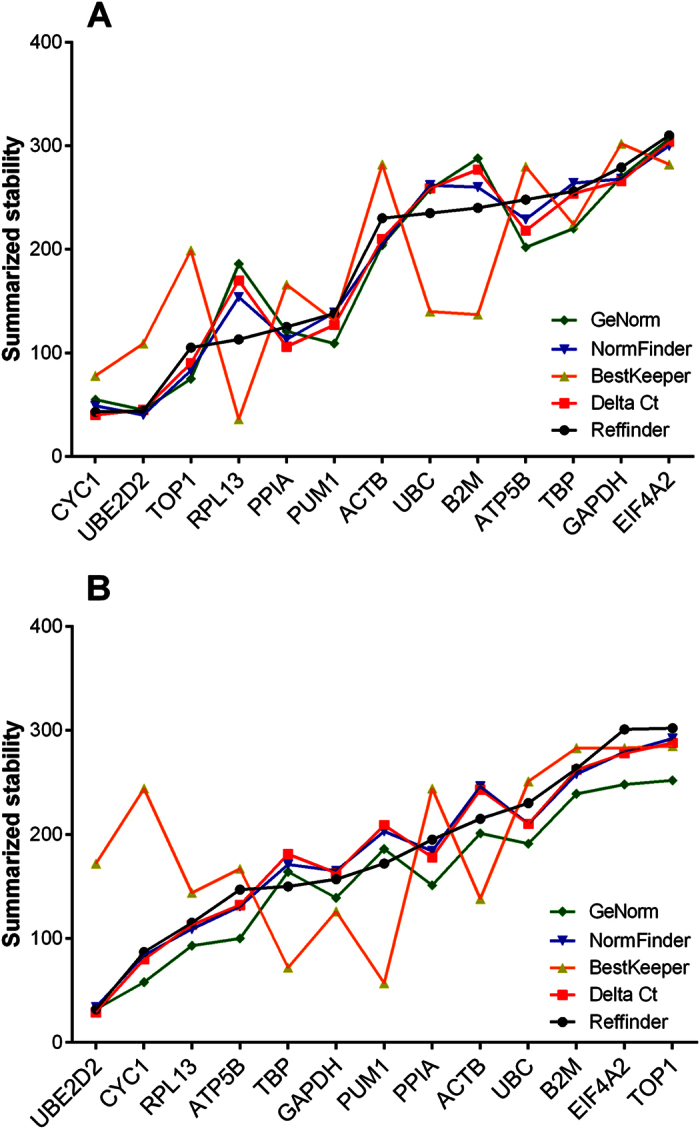
Summarized gene stability ranking for all individuals in the prefrontal cortex (**A**) and the cerebellum (**B**). Summarized rankings from RefFinder. Lower ranking indicates higher stability.

**Figure 3:**
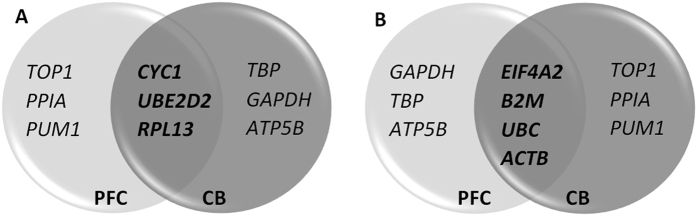
Ven diagram showing the overlap of the six most stable (**A**) and seven least stable (**B**) reference genes in both the prefrontal cortex (light gray) and the cerebellum (dark gray). The ven diagram is based on the summarized comprehensive rankings from RefFinder.

Based on the outcome from the RefFinder software (for all results, see Excel S1), candidate RGs were assigned a rank from 1 to 13 (1 being the most stable RG in the given combination of disease groups and 13 being the least stable; Excel S1). When integrating all different combinations for all disease groups included in the study, we found that in both brain regions *CYC1* and *UBE2D2* were the most stable RGs (Fig. 2). The least stable RG candidates in the PFC were *GAPDH* and *EIF4A2,* while *EIF4A2* and *TOP1* were ranked the least stable in the CB. Further, the summarized, comprehensive rankings for both brain regions and all individuals were considered collectively. *CYC1*, *UBE2D2*, and *RPL13* were ranked among the top six most stable RGs as illustrated in Fig. 3A. Of the seven most unstable RGs, *EIF4A2*, *B2M*, *UBC*, and *ACTB* were identified in both the PFC and CB (Fig. 3B). The overall three most stable RGs identified in this study are *CYC1*, *UBE2D2*, and *RPL13* (Fig. 3).

### Reference gene validation

**Figure 4:**
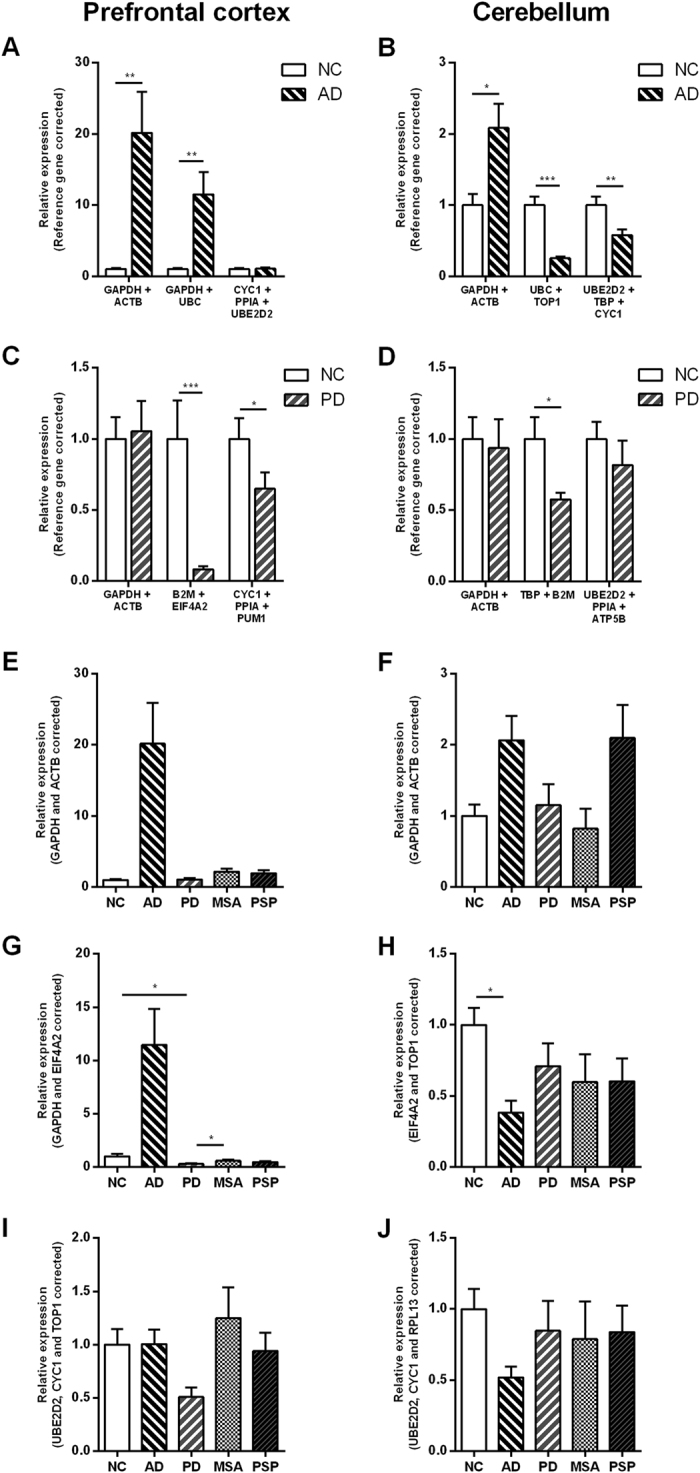
Expression levels of *GSK3B* normalized to different reference genes (RGs). *GSK3B* expression levels were analyzed using the common RG choices (*GAPDH* and *ACTB*) in combination, using the two lowest ranked genes in combination, and using the three best ranked genes in combination. (**A)** and **B**) shows comparison of RG choice for normal, non-demented controls (NCs) and Alzheimer’s disease (AD) patients, (**C)** and (**D**) for NCs and Parkinson’s disease (PD) patients, (**E–H**) for all groups. (**A**,**C**,**E**,**G)** and (**I**) show comparisons for the prefrontal cortex, and (**B**,**D**,**F**,**H**) and (**J**) for the cerebellum. All data are presented as the relative expression levels normalized to the most stable RGs calculated for each combination of disease groups in the respective areas, and data are shown in relation to expression levels in NCs. MSA: Multiple System Atrophy patients; PSP: Progressive Supranuclear Palsy patients. Data are presented as mean ± SEM. *p < 0.05, **p < 0.01, ***p < 0.001.

In order to demonstrate the impact of the RG choice on RT-qPCR data normalization, target gene *GSK3B* expression levels were analysed in both the PFC and the CB, between NCs and AD (Fig. 4A,B), NCs and PD (Fig. 4C,D), as well as between all groups (Fig. 4E–J). For each combination of disease groups, data were normalized to three distinct sets of RGs: 1) *GAPDH* and *ACTB*, the two most commonly used RGs; 2) the two RG candidates that in our analyses displayed the lowest stability for the given combination of disease groups; and 3) the three most stable RG candidates that in our analysis displayed the highest stability for the given combination of disease groups. The gene expression levels measured for *GSK3B* were significantly influenced by the choice of RGs used for normalization (Fig. 4, summarized in Table 3).

The greatest effect on the results is seen for the analysis of data from NCs vs AD. The expression of *GSK3B* is significantly upregulated in AD when normalized to the most common RGs and the least stable RGs in the PFC (Fig. 4A), and to the most common RGs in the CB (Fig. 4B). However, when we normalized the *GSK3B* expression levels to the most stable RGs obtained by our analyses, the relative significance between the groups disappeared in PFC (Fig. 4A) or is swapped in CB (Fig. 4B). For NCs vs PD normalization to the most common, and the most stable RGs resulted in similar outcomes, however, SDs when using the most stable RGs were decreased (Fig. 4C,D). When all groups were analysed together and *GSK3B* levels were normalized to the most stable RGs we observed the most equal, least diverging results between groups (Fig. 4I,J). A high increase of *GSK3B* levels in the AD group vs. other groups was observed in the PFC for data normalized to both the least stable RGs (Fig. 4G) and the most common RGs (Fig. 4E). Conversely, decreased levels of *GSK3B* were observed in the AD patients compared to NCs when the least stable RGs were used for data normalization in the CB (Fig. 4H), whereas no differences were identified in the CB for the most stable RGs (Fig. 4J).

### Correlation analyses

Significant differences in RNA integrity number (RIN) values, age, and post-mortem intervals (PMIs) were observed between groups (Table 4). To evaluate the impact of these factors on expression of *GSK3B*, raw Ct-values, and Ct-values normalized to the most stable RGs were correlated to RIN-values, age, and PMIs (Table S3). None of the viable factors showed significant correlation to the expression of *GSK3B*.

## Discussion

Our study is the first to provide a comprehensive evaluation of commonly used RGs for RT-qPCR for comparative studies of four neurodegenerative diseases: Two highly investigated diseases, AD and PD, along with two rare diseases, MSA and PSP. There is a general consensus on the use of HKGs as RGs, however, it has become more evident that the assumption of RG stability is not consistent when experimental conditions are altered. Therefore, we agree with others that, optimally, selection of the most valid RGs should be performed individually for each experimental setup^31^ as well as the investigated tissue, organ and region, disease, species and so forth^11^. Additional factors such as primer efficiencies, primer sequences, cDNA synthesis protocols, and basic experimental differences could also influence the outcome in RG stability, and therefore should also be considered. Several other factors have also been shown to influence the output of mRNA studies including degree of degradation^32^,^33^, PMIs^34^, and subject age^35^. As suggested by the minimum information for publication of quantitative real-time experiments (MIQE) guidelines^36^ RNA fragmentation should be obtained for every mRNA sample used for RT-qPCR. One way to obtain this is by determining the RIN-values^37^. It is important to obtain RIN-values as high as possible, as done in this study, with a threshold of 3.95 recommended for human post-mortem studies^38^. However, short amplicons (below 250 base pairs) are less dependent on RIN-values^33^. Further, some of the problems associated with low RIN-values can be circumvented using internal RGs for data normalization together with expressing result values relative to an internal standard (the ΔΔCt method^39^) with correction for PCR efficiency^32^. RIN-values have been shown to correlate negatively with PMIs^34^, however, we did not identify an influence of either PMIs or age on RIN-values (Fig. S2). We further corroborated that the raw or normalized Ct-values for *GSK3B* expression levels did not correlate with any of those factors in both brain regions. Thus, we can conclude that by applying the recommendations from the MIQE guidelines, and by implementing the precautions in the experimental design as described here, we were able to perform relative quantification of gene expression levels with a minimum risk of false interpretation of the results.

As mentioned above, PMIs varied significantly between groups. The samples used in this study originated from three different brain banks placed in Denmark, the Netherlands, and the USA. The variations in PMIs are primarily due to differences in national legislations regarding PMIs after which the extraction of human brains is allowed, and this is therefore difficult to circumvent. Since our findings did not show any significant correlations between PMI and RIN, age, raw Ct-values, or normalized Ct-values, this study moreover supports that inclusion of human brain samples from different sources is feasible even in the case of large differences in PMIs.

We applied the RefFinder software in this study for data analysis. The software combines four of the most commonly used algorithms for determination of the stability of gene expression (Genorm, NormFinder, BestKeeper and the ΔCt-method), but RefFinder does not share all of the features that are included in the stand-alone applications. E.g., the capability of Genorm to define the optimal number of RGs using a predetermined cut-off value that determines how much adding another RG to the analyses affects the grouped stability of the best ranked RGs^16^. However, as already stated elsewhere^16^,^40^, using a minimum of three RGs should be appropriate, and we therefore do not consider the absence of this feature in RefFinder to be of relevance for our analysis. RefFinder does not take primer efficiencies into account, a feature included in the Genorm, NormFinder and BestKeeper stand-alone applications. In order to correct for this, we manually adjusted the raw Ct-values for efficiencies before analysis. This has previously proved to make results obtained with the RefFinder software comparable to those obtained with the stand-alone applications^22^. Similar and comparable evaluations are obtained from the Genorm, NormFinder, and the ΔCt-methods, with deviations for some genes obtained from BestKeeper. This is surprising as earlier findings showed equal stability rankings for Genorm, NormFinder, and BestKeeper^21^,^22^. An explanation could be the so-called BestKeeper index, a tool that only BestKeeper applies. This index is the geometric mean of the Ct-values of all candidate RGs grouped together. Thus, the gene with the highest coefficient of correlation with the index indicates the highest stability. Despite of these small discrepancies, results regarding the best RG candidates were consistent between the different applied methods.

In this study we have used different approaches to assess RG candidate stabilities. Had we only used descriptive statistics with the only criteria applied being minimal variation in the expression levels in each group followed by low differences between disease groups, *TBP*, *PUM1*, and *RPL13* would be the preferable choices in both the PFC and CB in this study. Although *RPL13* ranks among the four most stable RGs according to the summarized rankings, *TBP* ranks among the three least stable RGs and it would therefore be inadvisable to use, whereas *PUM1* seems to be an intermediate RG. Thus, in order to evaluate candidate RGs based on a wide range of equally important parameters more advanced methodical and statistical approaches are needed.

According to our analyses using RefFinder, *CYC1*, *UBE2D2*, and *RPL13* were ranked among the top six most stable RGs, while *EIF4A2*, *B2M*, *UBC*, and *ACTB* were among the most unstable RGs. CYC1 and UBE2D2 proteins are affected in at least AD and/or PD^41^,^42^,^43^, but this is apparently irrelevant to the gene expression stability. The CYC1 protein is part of the mitochondrial electron transport chain and is thus crucial for cellular respiration^44^. UBE2D2 protein is a ubiquitin-conjugating enzyme that has been proposed as a general HKG along with proteins with similar functions^45^. Our results for *UBE2D2* find support elsewhere^27^. To our knowledge, neither gene transcription nor protein levels of the ribosomal 60S-subunit RPL13 have been reported to be affected in any of the diseases included in this study.

As suspected, *GAPDH* and *ACTB* did not show up as stable RGs in our analyses. GAPDH protein is a catalytic enzyme involved in glycolysis and is therefore important for cell metabolism^46^. Most likely due to this vital function, *GAPDH* has been one of the primary choices as RG in RT-qPCR studies for more than two decades. However, GAPDH protein and its activity has been shown to be affected in AD^8^ and PD^9^, and general *GAPDH* transcription levels have been shown to vary highly between different tissue compartments^47^. Our data indicate that *GAPDH* is not a stable RG in neither the PFC (ranked 12^th^) nor the CB (ranked 6^th^). ACTB protein is one of six different human actin isoforms and is one of the two non-muscle cytoskeletal actins^48^. *ACTB* has also been used extensively as an RG for several years. In our studies *ACTB* is ranked 7^th^ and 9^th^ in the PFC and CB, respectively, and it is therefore not recommended for use as a RG in similar experimental setups.

Finally, in order to validate how the choice of RGs influences RT-qPCR results, we compared *GSK3B* mRNA levels between different combinations of disease groups. *GSK3B* is regulating several different cellular processes, and *GSK3B* dysregulation have been implicated in the pathogenesis of both AD and PD^49^. Substantial divergences occurred in the relative transcript abundance of *GSK3B* when normalized to different sets of reference genes. Generally, large variations in the relative expression of *GSK3B* are seen when the most common, *GAPDH* and *ACTB*, or the least stable RGs are used for normalization. Conversely, when the most stable RGs are used, relative differences in mRNA levels between the groups become minimized. Bearing in mind that *GAPDH* and *ACTB* proved to be unstable RGs, the spread between these observations confirm the importance of using the adequate RGs in order to avoid false results.

In summary, the expression profiles and stability of 13 commonly used RGs in brain samples from four different neurodegenerative disorders (AD, PD, MSA and PSP) and NCs in two distinct brain regions (PFC and CB) were investigated using six different statistical approaches (descriptive statistics, Genorm, NormFinder, BestKeeper, ΔCt-method, and RefFinder). Furthermore, the relative expression of the disease associated gene *GSK3B* was assessed using different sets of RGs. This approach validated the impact that the choice of RGs has on the study outcome and underlined the importance of selecting the most stable RGs to correctly quantify gene expression levels. We suggest performing thorough analyses on RG selection for each tissue, disease, and experimental setup prior to the main experiment. Based on the results from this study we recommend using *UBE2D2, CYC1,* and *RPL13* in combination for studies related to brain tissue and to the diseases included here. Further, we have provided several important considerations regarding the choice and the assessment of RGs in RT-qPCR studies. This report should therefore be regarded as a guideline on how to perform RG validation in order to reinforce the reliability of RT-qPCR results.

## Methods

### Source of human brain tissue

Post-mortem brain samples from the medial frontal gyrus of the PFC and the CB (Tables S1 and S2) were generously donated by the Brain Bank at Bispebjerg-Frederiksberg Hospital (University of Copenhagen; approved by the Danish Data Protection Agency, j.no. BBH-2010-06, I-suite 00971), the Netherlands Brain Bank (Netherlands Institute for Neuroscience), and the Harvard Brain Tissue Resource Center (Harvard Medical School Teaching Hospital) (Table S1). All samples were histologically investigated to confirm pathology and diagnosis. A significant difference in age in the MSA group is observed due to their earlier disease onset and death (Table 4). All brain samples were collected, and handled in accordance with Danish ethical standards, and the Danish Health and Medicine Authorities. This project was approved by the Regional ethical committee of Region Hovedstaden, journal no. H-16025196. Informed consent was obtained from all donors. Samples were stored at −80 °C prior to total RNA extraction.

### RNA isolation and cDNA synthesis

The RNA isolation and the subsequent RT-qPCR reactions were performed according to the MIQE guidelines^36^. Total RNA extraction was performed using either Qiagen RNeasy Lipid Tissue Mini Kit or Qiagen AllPrep DNA/RNA/miRNA Universal Kit according to manufacturer’s instructions. Prior to RNA extraction, brain samples were homogenized using a MagNA Lyser instrument (Roche Diagnostics) and the related MagNA Lyser green beads (Roche) at 2 × 6.5 k rpm for 25 seconds in 1 ml pre-cooled Qiazol reagent supplied with the extraction kit. RNA quality and concentrations were assessed using the 2100 Agilent Bioanalyzer (Agilent Technologies) using RNA 6000 Nano kits (Agilent Technologies) following the manufacturer’s instructions. RNA purity was assessed using a NanoDrop 2100 (Thermo Scientific). RNA concentrations ranged from 0.04–1.10 μg/μl and RIN-values from 4.0–7.8. A cut-off threshold of RIN = 3.95 based on^34^,^38^, and an optical density at wavelength 260/280 nm range of 1.8–2.2 were chosen for this study^36^. All RNA samples were subjected to DNase digestion using the Turbo DNA-*free*^TM^ Kit (Ambion) and subsequently tested for DNA contamination in RT-qPCR using the *GAPDH* primer set (Table 1). Single-stranded cDNA was synthesized from 100 ng of total RNA using qScript cDNA SuperMix kit (Quanta) following the manufacturer’s instructions. cDNA concentrations were measured on a NanoDrop 2000 and diluted to 100 ng/μl in RNase-free H_2_O.

### Selection of candidate reference genes and PCR primer design

15 putative RGs were selected for evaluation of their stability in each patient group and in comparisons of patient groups. The RGs were selected based on their vast usage in the literature and in commercial gene array kits. Six primer pairs were adapted from the literature: *ATP5B*^50^, *TOP1*^51^, *PPIA*^52^, *PUM1*^53^, *TBP* and *UBE2D2*^54^. For all other genes primer pairs were designed *de novo* using Primer-BLAST (NCBI) and Oligo 7 (Molecular Biology Insight, Inc.). Primers were synthesized by TAG Copenhagen A/S. For each primer pair (Table 1), the optimal annealing temperature, the amplification efficiency, and *R*^*2*^ were determined and calculated using the MxPro software package (Agilent Technologies).

### Reverse Transcriptase Semi-Quantitative Real-time PCR (RT-qPCR)

All samples were run in duplicates. Briefly, RT-qPCR reactions were carried out using Fast SYBR Green Master Mix (Applied Biosystems) on a Stratagene Mx3005p qPCR System (Agilent Technologies). The final volume for each reaction was 10 μl with 300 nM (400 nM for *TOP1*) of corresponding gene specific primers (Table 1), and 1 μl of total cDNA. A positive control/calibrator cDNA sample synthesized from commercial available Human Universal Reference Total RNA (hRNA; Clontech) was included on each plate. A negative water control was included in each run. The thermal cycling was initiated at 95 °C for 20 s followed by 40 cycles of 5 s at 95 °C and 30 s at the optimal annealing temperature for each gene (Table 1). If primer dimers were observed, an additional acquisition step for 15 s in each cycle was added to avoid primer dimers detection (Table 1 and Fig. S1). Dissociation curve analyses were carried out at the end of each run for PCR product verification (Fig. S1).

### Data analysis

The stabilities of the RG candidates were accessed using four methods: Genorm^16^, NormFinder^18^, BestKeeper^17^ and the comparative ΔCt method^15^ combined in the online package RefFinder which includes an overall comprehensive analyses of the four methods^20^ (http://fulxie.0fees.us). The mean Ct-values for each sample were normalized using a Human Universal Reference cDNA as a calibrator as described by^39^. Furthermore, the Ct-values were corrected for the efficiencies for each gene. Relative quantities were calculated using geometric averaging of multiple reference genes^55^.

### Statistics

Data are displayed as mean ± SEM. Gaussian distribution was evaluated using the D’Agostino-Pearson omnibus normality test. Homogeneity of variance was evaluated using Levene’s test. Outliers were removed using the ROUT procedure with maximum false discovery rate (FDR) Q = 1%. Statistical analyses were conducted in GraphPad Prism 6.01 (GraphPad Software) and the Real Statistics Resource Pack software v. 4.8 using non-paired parametric Student’s *t*-test, Welch’s *t*-test for unequal variances, one-way ANOVA followed by Tukey’s multiple comparison range test, Welch’s ANOVA followed by Games-Howell post hoc test, and Bonferroni corrected Pearson product-moment correlation or Mann-Whitney *U* test, and Bonferroni corrected Spearman rank correlation. Chi-square test was used to assess the gender frequency. *p*-values below 0.05 were considered statistically significant.

## Additional Information

**Accession codes:** ATP5B (NM_001686), B2M (NM_004048), PPIA (NM_021130), CYC1 (NM_001916), EIF4A2 (NM_001967), GAPDH (NM_002046), PUM1 (NM_014676), RPL13 (NM_000977), TBP (NM_003194), TOP1 (NM_003286), UBC (NM_021009), UBE2D2 (NM_003339), ACTB (NM_001101), GSK3B (NM_002093).

**How to cite this article**: Rydbirk, R. *et al.* Assessment of brain reference genes for RT-qPCR studies in neurodegenerative diseases. *Sci. Rep.* **6**, 37116; doi: 10.1038/srep37116 (2016).

**Publisher’s note:** Springer Nature remains neutral with regard to jurisdictional claims in published maps and institutional affiliations.

## Supplementary Material

Supplementary Information

Supplementary Dataset 1

## Figures and Tables

**Table 1:** Overview of genes and corresponding primer pairs for reverse transcriptase quantitative real-time PCR.

Gene symbol	Accession no.	Primer sequence (5′-3′)		Bp	*E* (%)	*R*^*2*^	T_a_	T_aq_
*ATP5B*	NM_001686	Sense: Anti-sense:	TCACCCAGGCTGGTTCAGA AGTGGCCAGGGTAGGCTGAT	80	106.3	0.996	60	
*B2M*	NM_004048	Sense: Anti-sense:	ACTGAATTCACCCCCACTGA CCTCCATGATGCTGCTTACA	114	102.7	0.997	56	
*PPIA*	NM_021130	Sense: Anti-sense:	TCCTGGCATCTTGTCCATG CCATCCAACCACTCAGTCTTG	90	101.5	1.000	58	
*CYC1*	NM_001916	Sense: Anti-sense:	AGCCTACAAGAAAGTTTGCCTAT TCTTCTTCCGGTAGTGGATCTTGGC	126	104.3	1.000	58	80
*EIF4A2*	NM_001967	Sense: Anti-sense:	AATTCCGGTCAGGGTCAAGTC GCCACACCTTTCCTCCCAAA	166	102.0	0.994	56	
*GADPH*	NM_002046	Sense: Anti-sense:	CGCTCTCTGCTCCTCCTGTT CCATGGTGTCTGAGCGATGT	81	101.5	0.999	60	
*PUM1*	NM_014676	Sense: Anti-sense:	AGTGGGGGACTAGGCGTTAG GTTTTCATCACTGTCTGCATCC	111	97.8	0.997	58	
*RPL13*	NM_000977	Sense: Anti-sense:	CCTGGAGGAGAAGAGGAAAGAGA TTGAGGACCTCTGTGTATTTGTCAA	126	90.4	0.998	56	
*TBP*	NM_003194	Sense: Anti-sense:	GCCCGAAACGCCGAATATAA AATCAGTGCCGTGGTTCGTG	81	100.2	1.000	58	
*TOP1*	NM_003286	Sense: Anti-sense:	GGCGAGTGAATCTAAGGATAATGAA TGGATATCTTAAAGGGTACAGCGAA	97	99.5	0.992	56	
*UBC*	NM_021009	Sense: Anti-sense:	CACTTGGTCCTGCGCTTGA TTATTGGGAATGCAACAACTTTAT	104	101.3	0.993	56	
*UBE2D2*	NM_003339	Sense: Anti-sense:	TGCCTGAGATTGCTCGGATCT TCGCATACTTCTGAGTCCATTCC	81	107.6	0.999	58	
*ACTB*	NM_001101	Sense: Anti-sense:	TGACATTAAGGAGAAGCTGTGCTAC ACTTCATGATGGAGTTGAAGGTAGT	224	104.0	0.990	60	82
*GSK3B*	NM_002093	Sense: Anti-sense:	ACAACAGTGGTGGCAACTCC TTCTTGATGGCGACCAGTTCT	146	93.0	0.989	60	

*R*^*2*^ and the primer efficiency*, E*, was calculated using at least four points on the standard curve. Bp: length of the amplicon in base pair; T_a_: Annealing temperature [°C]; T_aq_: Acquisition temperature [°C].

**Table 2:** Mean raw Ct-values and standard deviations (SDs) for each disease group and controls in both the prefrontal cortex, and the cerebellum.

Gene	Region	NC	*SD*	AD	*SD*	PD	*SD*	MSA	*SD*	PSP	*SD*	Average	
		*Mean*		*Mean*		*Mean*		*Mean*		*Mean*		*Ct*	*SD*
*ACTB*	PFC	26.9	2.67	26.7	1.46	28.9	2.22	29.4	1.47	29.6	1.68	28.3	1.90
	CB	29.8	1.42	29.1	1.26	29.9	0.68	30.3	1.21	32.4	2.31	30.3	1.38
*ATP5B*	PFC	27.6	2.68	26.9	0.89	29.9	1.46	30.5	1.25	30.0	1.75	29.0	1.61
	CB	29.9	1.27	28.0	1.34	29.7	1.07	29.3	1.57	31.0	2.03	29.6	1.46
*B2M*	PFC	30.9	3.09	29.9	1.30	29.3	1.35	30.8	1.77	30.7	0.96	30.3	1.69
	CB	30.5	1.32	28.0	1.61	29.9	1.11	28.9	1.26	31.0	1.98	29.6	1.46
*PPIA*	PFC	24.7	2.40	23.9	1.15	26.3	1.07	27.0	1.09	26.3	1.26	25.7	1.39
	CB	27.5	0.79	24.7	1.26	27.0	0.96	26.8	1.37	29.5	2.50	27.1	1.38
*CYC1*	PFC	26.8	2.25	26.3	1.35	27.9	1.02	28.6	1.05	28.1	1.20	27.6	1.37
	CB	27.8	1.11	24.9	1.11	27.4	1.05	27.3	1.44	29.3	2.34	27.3	1.41
*EIF4A2*	PFC	33.6	3.09	32.5	2.31	31.7	1.64	32.8	1.99	32.5	1.85	32.6	2.18
	CB	31.1	1.49	28.5	1.88	30.5	1.18	30.3	1.92	31.4	2.51	30.4	1.80
*GADPH*	PFC	26.2	2.45	31.9	3.07	28.1	1.49	28.8	1.28	28.4	1.77	28.7	2.01
	CB	27.1	1.32	26.1	1.21	26.7	1.03	26.7	1.33	28.9	1.94	27.1	1.37
*PUM1*	PFC	29.3	1.97	28.4	1.25	30.9	1.01	31.5	0.90	31.3	1.07	30.3	1.24
	CB	33.5	1.09	31.5	0.66	32.7	0.95	32.4	1.10	34.1	1.47	32.8	1.05
*RPL13*	PFC	24.8	1.95	24.3	1.80	24.7	0.91	25.4	0.89	25.4	1.12	25.0	1.33
	CB	24.1	1.20	21.3	0.96	23.4	0.90	23.7	1.33	24.9	1.79	23.5	1.24
*TBP*	PFC	29.9	1.84	29.2	1.32	32.6	0.97	33.3	0.81	32.7	1.09	31.5	1.21
	CB	31.5	1.03	29.0	1.13	31.2	1.21	30.7	1.07	32.0	1.03	30.9	1.09
*TOP1*	PFC	29.8	2.55	28.7	1.73	30.1	1.13	31.3	1.28	30.6	1.47	30.1	1.63
	CB	32.4	1.04	28.0	0.68	31.4	0.87	31.4	1.65	32.0	2.14	31.0	1.28
*UBC*	PFC	29.3	2.55	28.1	2.88	28.1	1.06	28.9	0.93	28.3	1.14	28.5	1.71
	CB	28.0	1.02	24.7	1.09	27.7	1.28	28.0	1.17	29.9	1.96	27.7	1.30
*UBE2D2*	PFC	29.5	2.31	28.8	1.54	30.1	0.98	31.0	0.99	30.2	1.30	29.9	1.42
	CB	30.4	0.99	27.5	1.16	29.9	0.76	29.7	1.52	31.5	1.81	29.8	1.25

NC: Normal controls; AD: Alzheimer’s disease; PD: Parkinson’s disease; MSA: Multiple System Atrophy; PSP: Progressive Supranuclear Palsy; PFC: Prefrontal cortex; CB: Cerebellum. Means are geometric.

**Table 3:** Results from *GSK3B* expression comparison.

Groups	Most common RGs		Least stable RGs		Most stable RGs	
	% of NC	p-value^1^	% of NC	p-value^1^	% of NC	p-value^1^
PFC						
NC vs AD^¤^	2015.9	0.009	1150.9	0.009	107.2	0.741
NC vs AD^¤^	105.3	0.839	8.2	<0.001	64.9	0.022
All groups^+^	—	0.004	NC&MSA > PD*	0.001	—	0.015
CB						
NC vs AD^¤^	208.6	0.012	25.3	<0.001	57.6	0.010
NC vs AD^¤^	93.5	0.660	57.5	0.023	81.7	0.245
All groups^+^	—	0.080	NC > AD*	0.043	—	0.383

*GSK3B*: Glycogen synthase kinase-3beta; RG: Reference gene; NC: Normal control; AD: Alzheimer’s disease; PD: Parkinson’s disease; MSA: Multiple System Atrophy. ^1^p-values for the t-test or the ANOVA test are listed. Data were analyzed using: ^¤^Welch’s t-test or Student’s t-test; ^+^Welch’s weighted ANOVA followed by Games-Howell post-hoc test (^*^p < 0.05).

**Table 4:** Main clinical and neuropathological data for patient groups in the prefrontal cortex, and the cerebellum.

Region	Patient group	Origin	N	Braak stage	Sex	Age [years]	PMI [hours]	RIN
**PFC**	NC	NBB	10	1.3 ± 0.5	4M/6F	81.0 ± 9.5	9.2 ± 5.8	5.1 ± 0.6
	AD	NBB	10	4.7 ± 0.7	4M/6F	81.9 ± 8.5	4.7 ± 1.1	5.0 ± 0.6
	MSA	BBH	10	—	4M/6F	64.4 ± 6.8	41.6 ± 24.1	5.1 ± 0.7
	PD	HV	10	—	7M/3F	79.5 ± 6.0	18.3 ± 6.9	5.8 ± 0.9
	PSP	7BBH/3HV	10	—	8M/2F	72.4 ± 8.7	30.1 ± 15.1	5.8 ± 1.0
	p-value			**0.0001**	0.190*	** < 0.001**	** < 0.001**	**0.055**
**CB**	NC	BBH	10	—	4M/6F	72.1 ± 7.8	36.3 ± 18.0	6.7 ± 0.5
	AD	NBB	10	4.7 ± 0.7	5M/5F	78,6 ± 7.6	5.1 ± 2.5	6.1 ± 1.1
	MSA	BBH	8	—	2 M/6 F	63.9 ± 7.7	43.8 ± 16.9	5.4 ± 0.8
	PD	HV	10	—	8 M/2 F	78.1 ± 7.2	17.8 ± 5.5	6.1 ± 0.5
	PSP	7BBH/3HV	10	—	8 M/2 F	74.9 ± 8.1	33.0 ± 17.0	6.0 ± 1.1
	p-value			—	0.061*	**0.002**	** < 0.001**	**0.036**

NC: Normal control; AD: Alzheimer’s disease; PD: Parkinson’s disease; MSA: Multiple System Atrophy; PSP: Progressive Supranuclear Palsy; PFC: Prefrontal cortex; CB: Cerebellum; NBB: Netherlands Brain Bank; BBH: Bispebjerg-Frederiksberg University Hospital Brain Bank; HV: Harvard Brain Tissue Resource Center; M: male; F: female; RIN: RNA integrity number. *p*-values: One-Way ANOVA followed by Tukey’s post-hoc test. ^*^Chi-Squared test of independence.
