# Author Correction: Assessment of brain reference genes for RT-qPCR studies in neurodegenerative diseases

**DOI:** 10.1038/s41598-020-68129-5

**Published:** 2020-07-23

**Authors:** Rasmus Rydbirk, Jonas Folke, Kristian Winge, Susana Aznar, Bente Pakkenberg, Tomasz Brudek

**Affiliations:** 1 Research Laboratory for Stereology and Neuroscience, Bispebjerg-Frederiksberg Hospital, University Hospital of Copenhagen, Bispebjerg Bakke 23, DK-2400 Copenhagen, Denmark; 2 Bispebjerg Movement Disorder Biobank, Bispebjerg-Frederiksberg Hospital, University Hospital of Copenhagen, Bispebjerg Bakke 23, DK-2400 Copenhagen, Denmark; 3 Department of Neurology, Bispebjerg-Frederiksberg Hospital, University Hospital of Copenhagen, Bispebjerg Bakke 23, DK-2400 Copenhagen, Denmark; 4 Institute of Clinical Medicine, Faculty of Health, University of Copenhagen, Blegdamsvej 3B, DK-2200 Copenhagen, Denmark

Correction to: *Scientific Report* 10.1038/srep37116, published online 17 November 2016

This Article contains errors.

In this study, the Authors have used two different primer sets for the gene *RPL13*. However, as a result of an error during preparation of the manuscript, one of these sets was labelled as *CYC1*. The changes to the labelling have resulted in the following corrections to the Article. Although both primer sets for *RPL13* are among most stable, the Authors recommend that only one set is used for normalisation as it is inadvisable to use two primer sets for the same gene.

In the Abstract,

“Using RefFinder, a web-based tool for evaluating RG stability, we identified the most stable RGs to be UBE2D2, CYC1, and RPL13 which we recommend for future RT-qPCR studies on human brain tissue from these patients.”

should read:

“Using RefFinder, a web-based tool for evaluating RG stability, we identified the most stable RGs to be UBE2D2 and RPL13 which we recommend for future RT-qPCR studies on human brain tissue from these patients”.

In the Introduction,

“The following genes were investigated (Table 1): GAPDH, ACTB, ribosomal protein large 13 (RPL13), hypoxanhine phosphoribosyl transferase (HPRT1), cytrochrome C1 (CYC1), topoisomerase 1 (TOP1), eukaryotic translation initiation factor 4A2 (EIF4A2), β-2-microglobulin (B2M), pumilio-homolog 1 (PUM1), TATA-box binding protein (TBP), ubiquitin C (UBC), cyclophilin A (PPIA), succinate dehydrogenase complex subunit A (SDHA), ATP synthase H+ transporting mitochondrial F1 complex beta polypeptide (ATP5B), and ubiquitin-conjugating enzyme E2D2 (UBE2D2).”

should read:

“The following genes were investigated (Table 1): GAPDH, ACTB, ribosomal protein large 13 (RPL13) using two primer sets (RPL13(a) and RPL13(b)), hypoxanhine phosphoribosyl transferase (HPRT1), topoisomerase 1 (TOP1), eukaryotic translation initiation factor 4A2 (EIF4A2), β-2-microglobulin (B2M), pumilio-homolog 1 (PUM1), TATA-box binding protein (TBP), ubiquitin C (UBC), cyclophilin A (PPIA), succinate dehydrogenase complex subunit A (SDHA), ATP synthase H+ transporting mitochondrial F1 complex beta polypeptide (ATP5B), and ubiquitin-conjugating enzyme E2D2 (UBE2D2).”

In the Results, under the subheading ‘Descriptive statistics of candidate RGs’,

“Almost all candidate RGs showed significantly aberrant expression levels between the disease groups in both brain regions with the exception of RPL13, EIF4A2, B2M, and UBC in the PFC which showed no significant differences between groups (Fig. 1).”

should read:

“Almost all candidate RGs showed significantly aberrant expression levels between the disease groups in both brain regions with the exception of RPL13(b), EIF4A2, B2M, and UBC in the PFC which showed no significant differences between groups (Fig. 1).”

In the same section,

“The genes with the highest expression levels were in both brain regions *RPL13* (Ct_mean, PFC_ = 25.0 and Ct_mean, CB_ = 23.5), and *PPIA* (Ct_mean, PFC_ = 25.7 and Ct_mean, CB_ = 27.1). In all groups, *EIF4A2* showed the most variable expression levels in both regions reflected by the mean SD (SD_mean, PFC_ = 2.18 and SD_mean, CB_ = 1.80). In both brain regions, the RG candidates that exhibited the lowest variability in expression levels were *TBP* (SD_mean, PFC_ = 1.21 and SD_mean, CB_ = 1.09), *PUM1* (SD_mean, PFC_ = 1.24 and SD_mean, CB_ = 1.05), and *RPL13* (SD_mean, PFC_ = 1.33 and SD_mean, CB_ = 1.24).”

should read:

“The genes with the highest expression levels were in both brain regions *RPL13(b)* (Ct_mean, PFC_ = 25.0 and Ct_mean, CB_ = 23.5), and *PPIA* (Ct_mean, PFC_ = 25.7 and Ct_mean, CB_ = 27.1). In both brain regions, the RG candidates that exhibited the lowest variability in expression levels were *TBP* (SD_mean, PFC_ = 1.21 and SD_mean, CB_ = 1.09), *PUM1* (SD_mean, PFC_ = 1.24 and SD_mean, CB_ = 1.05), and *RPL13* (SD_mean, PFC_ = 1.33 and SD_mean, CB_ = 1.24).

In the Results, under the subheading ‘Summarized comprehensive ranking’,

“When integrating all different combinations for all disease groups included in the study, we found that in both brain regions *CYC1* and *UBE2D2* were the most stable RGs (Fig. 2)”

should read:

“When integrating all different combinations for all disease groups included in the study, we found that in both brain regions *RPL13* and *UBE2D2* were the most stable RGs (Fig. 2)”

In the same section,

“*CYC1*, *UBE2D2*, and *RPL13* were ranked among the top six most stable RGs as illustrated in Fig. 3A. Of the seven most unstable RGs, *EIF4A2*, *B2M*, *UBC*, and *ACTB* were identified in both the PFC and CB (Fig. 3B). The overall three most stable RGs identified in this study are *CYC1*, *UBE2D2*, and *RPL13* (Fig. 3)”

should read:

“*UBE2D2*, and *RPL13* were ranked among the top six most stable RGs as illustrated in Fig. 3A. Of the seven most unstable RGs, *EIF4A2*, *B2M*, *UBC*, and *ACTB* were identified in both the PFC and CB (Fig. 3B). The overall two most stable RGs identified in this study are *UBE2D2* and *RPL13* (Fig. 3).”

In the Discussion,

“Had we only used descriptive statistics with the only criteria applied being minimal variation in the expression levels in each group followed by low differences between disease groups, *TBP*, *PUM1*, and *RPL13* would be the preferable choices in both the PFC and CB in this study. Although *RPL13* ranks among the four most stable RGs according to the summarized rankings, *TBP* ranks among the three least stable RGs and it would therefore be inadvisable to use, whereas *PUM1* seems to be an intermediate RG.”

should read:

“Had we only used descriptive statistics with the only criteria applied being minimal variation in the expression levels in each group followed by low differences between disease groups, *TBP*, *PUM1*, and *RPL13(b)* would be the preferable choices in both the PFC and CB in this study. Although *RPL13(b)* ranks among the four most stable RGs according to the summarized rankings, *TBP* ranks among the three least stable RGs and it would therefore be inadvisable to use, whereas *PUM1* seems to be an intermediate RG.”

Additionally, in the Discussion,

“According to our analyses using RefFinder, *CYC1*, *UBE2D2* and *RPL13* were ranked among the top six most stable RGs, while *EIF4A2*, *B2M*, *UBC*, and *ACTB* were among the most unstable RGs. CYC1 and UBE2D2 proteins are affected in at least AD and/or PD^41,42,43^, but this is apparently irrelevant to the gene expression stability. The CYC1 protein is part of the mitochondrial electron transport chain and is thus crucial for cellular respiration^44^.”

should read:

“According to our analyses using RefFinder, *UBE2D2* and *RPL13* were ranked among the top six most stable RGs, while *EIF4A2*, *B2M*, *UBC*, and *ACTB* were among the most unstable RGs. UBE2D2 protein is affected in at least AD and/or PD^41,42,43^, but this is apparently irrelevant to the gene expression stability.”

Finally, in the Discussion,

“Based on the results from this study we recommend using *UBE2D2, CYC1,* and *RPL13* in combination for studies related to brain tissue and to the diseases included here.”

should read:

“Based on the results from this study we recommend using *UBE2D2* and *RPL13* in combination for studies related to brain tissue and to the diseases included here. Specifically, both primer sets for *RPL13* are the most stable according to our analyses. However, we find it inadvisable to use two primer sets for the same gene. Hence, we recommend using *RPL13(a)* for normalization since this primer set was even more stable than the *RPL13(b)* primer set.”

In the Additional Information,

“**Accession codes:** ATP5B (NM_001686), B2M (NM_004048), PPIA (NM_021130), CYC1 (NM_001916), EIF4A2 (NM_001967), GAPDH (NM_002046), PUM1 (NM_014676), RPL13 (NM_000977), TBP (NM_003194), TOP1 (NM_003286), UBC (NM_021009), UBE2D2 (NM_003339), ACTB (NM_001101), GSK3B (NM_002093)”

should read:

“**Accession codes:** ATP5B (NM_001686), B2M (NM_004048), PPIA (NM_021130), EIF4A2 (NM_001967), GAPDH (NM_002046), PUM1 (NM_014676), RPL13 (NM_000977), TBP (NM_003194), TOP1 (NM_003286), UBC (NM_021009), UBE2D2 (NM_003339), ACTB (NM_001101), GSK3B (NM_002093)”

In Table 1, “*CYC1*” should read “*RPL13(a)*”, “NM_01916” should read “NM_00977”, and “*RPL13*” should read “*RPL13(b)*”.

In Table 2, “*CYC1*” should read “*RPL13(a)*”, and “*RPL13*” should read “*RPL13(b)*”.

Labelling should be corrected in Figs. 1–4 and Supplementary Fig. 1, where “*CYC1*” should read “*RPL13(a)*”, and “*RPL13*” should read “*RPL13(b)*”. The corrected versions of these figures are included below as Figs. 1–5. Finally, the labels should also be corrected in the Supplementary Dataset. The corrected version of this file is included below.

**Figure 1:**
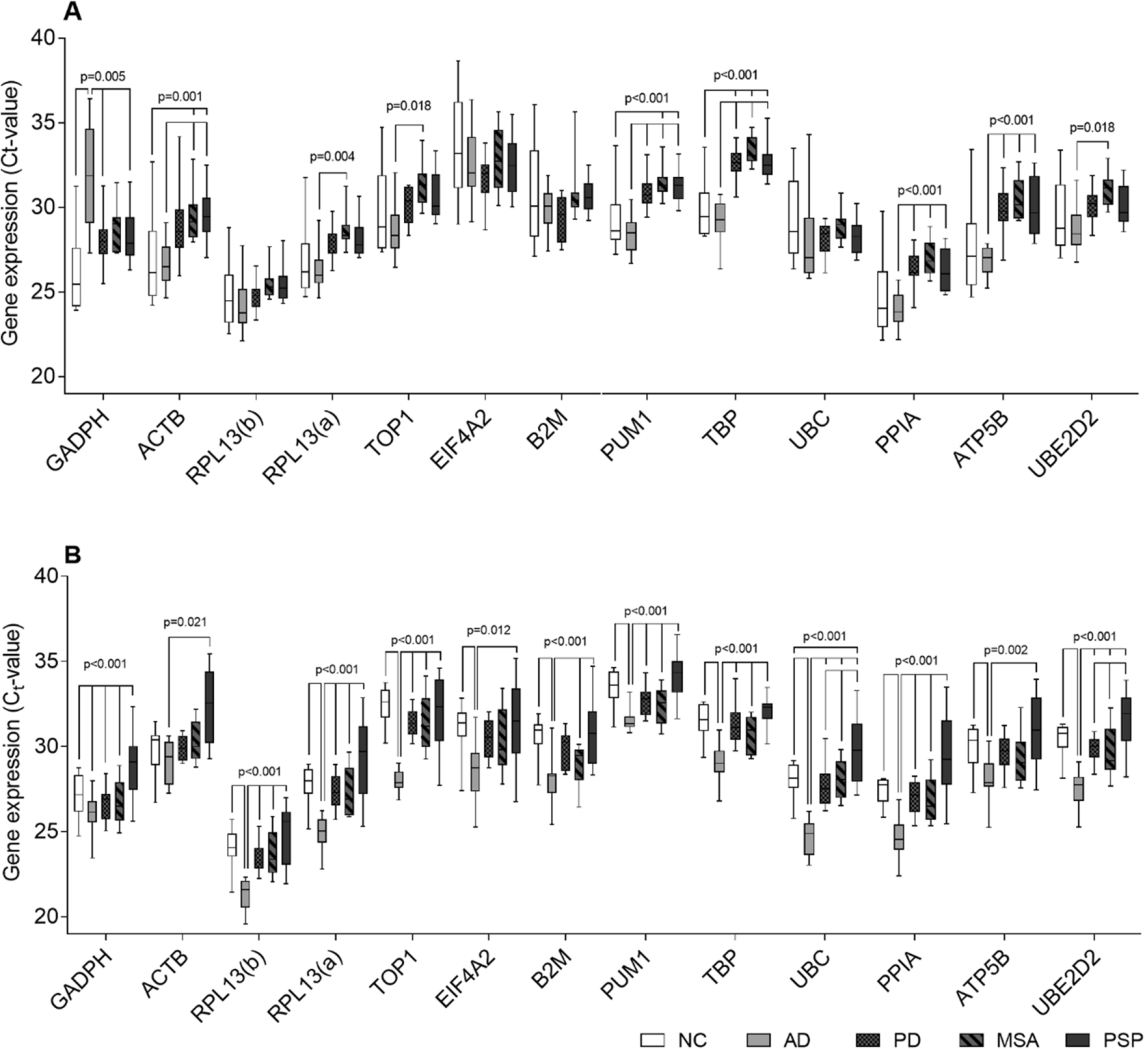
.

**Figure 2:**
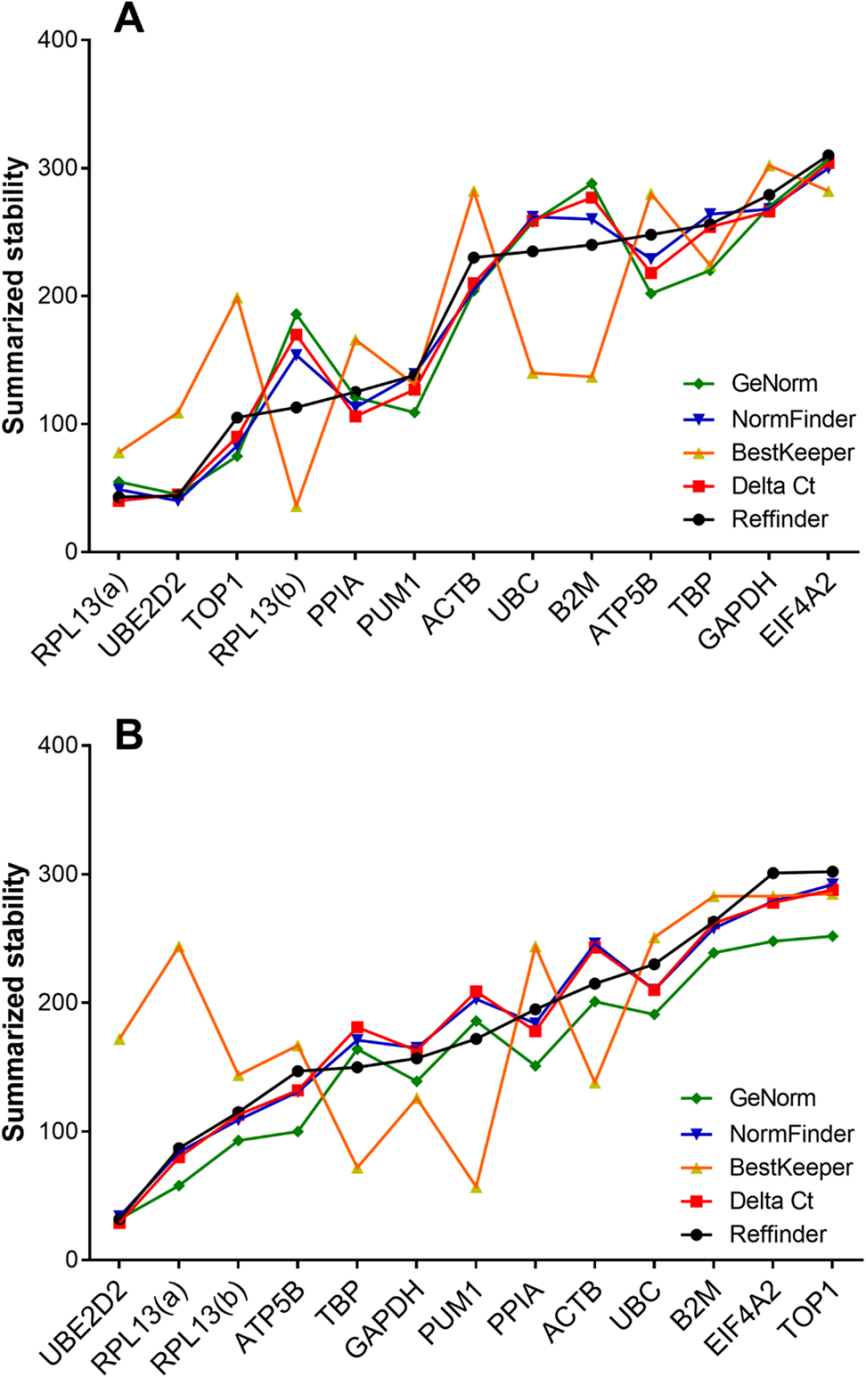
.

**Figure 3:**
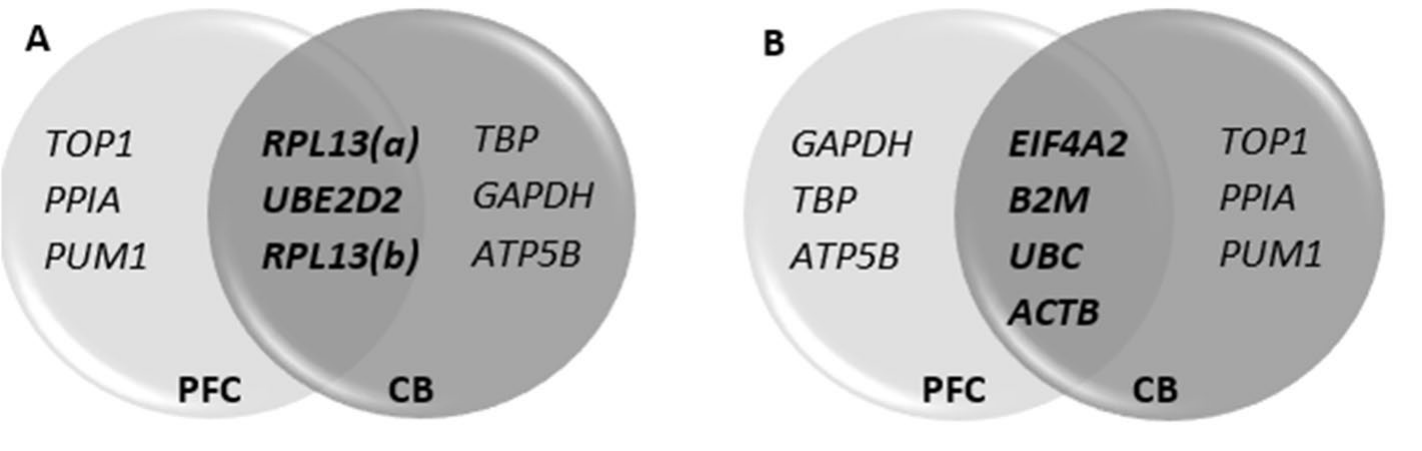
.

**Figure 4:**
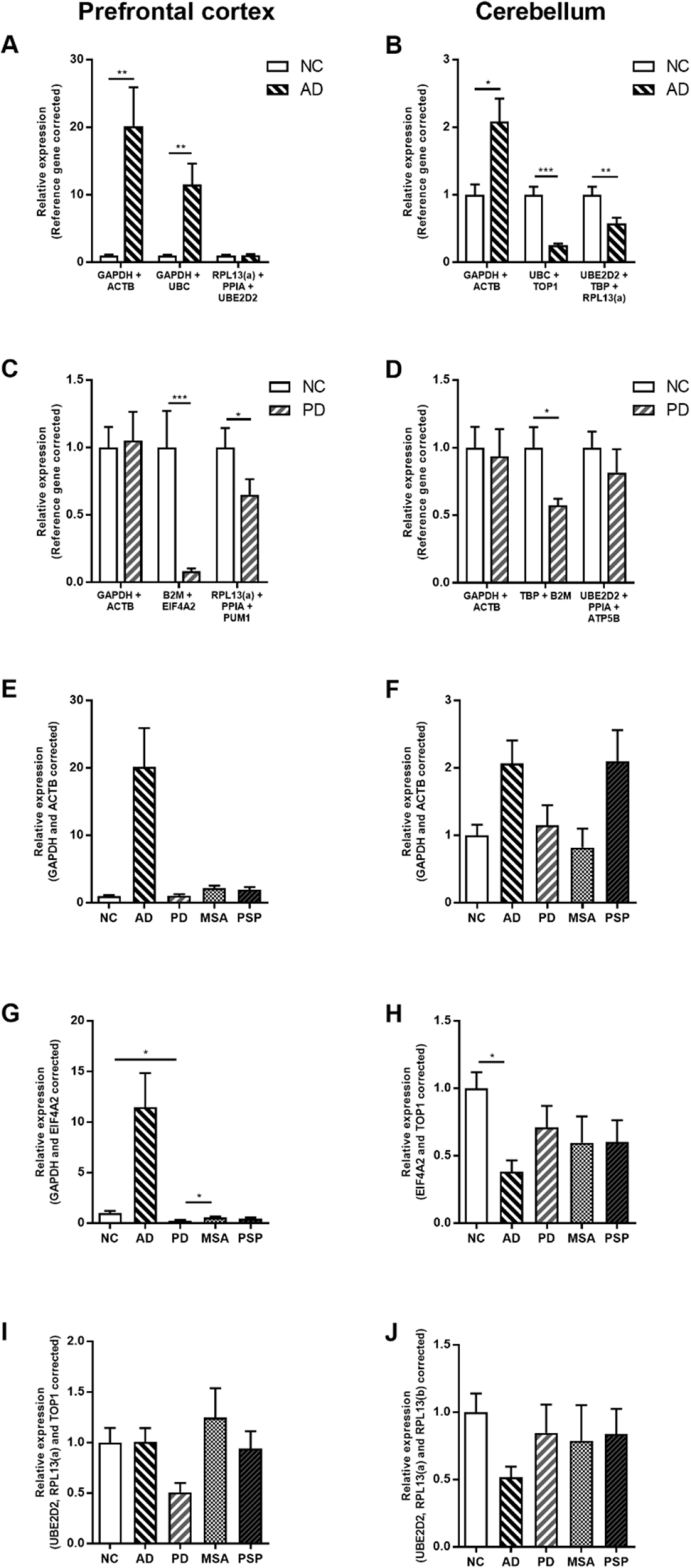
.

**Figure 5:**
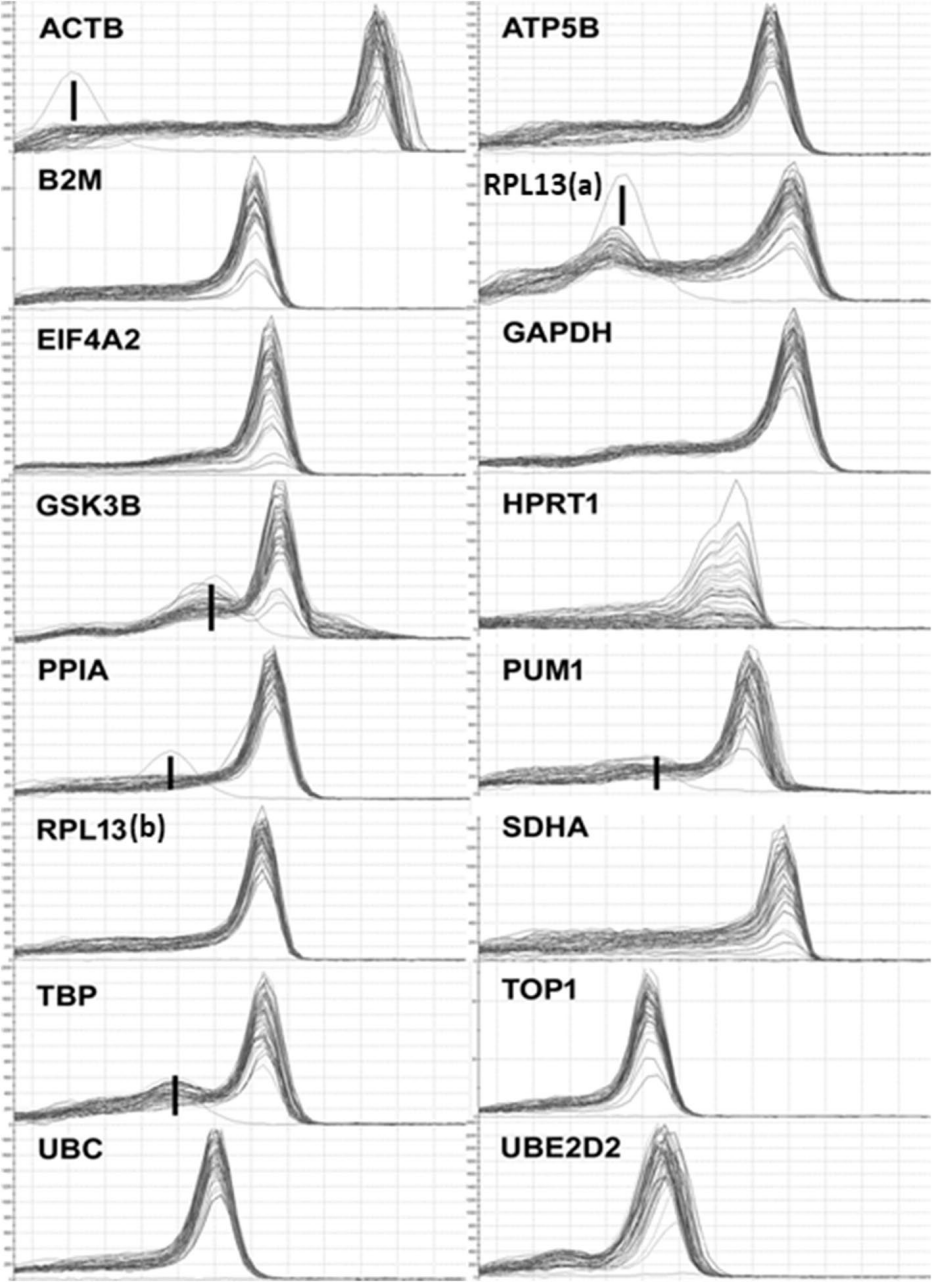
.

## Supplementary information

Supplementary information

