# Supplementary material for: Assessment of brain reference genes for RT-qPCR studies in neurodegenerative diseases
Source: Sci Rep. 2016 Nov 17;6:37116. doi: 10.1038/srep37116 (PMC5112547; doi:10.1038/srep37116)
Supplement: Supplementary Information [file srep37116-s1.pdf]

Title

Assessment of brain reference genes for RT-qPCR studies in neurodegenerative diseases

Authors and affiliations

Rasmus Rydbirk[i*], Jonas Folke[i], Kristian Winge[ii,iii], Susana Aznar[i], Bente Pakkenberg[i,iv] and Tomasz Brudek[i]

[i]Research Laboratory for Stereology and Neuroscience, Bispebjerg-Frederiksberg Hospital, University Hospital of Copenhagen, Bispebjerg Bakke 23, DK-2400, Copenhagen, Denmark

[ii]Bispebjerg Movement Disorder Biobank, Bispebjerg-Frederiksberg Hospital, University Hospital of Copenhagen, Bispebjerg Bakke 23, DK-2400, Copenhagen, Denmark

[iii]Department of Neurology, Bispebjerg-Frederiksberg Hospital, University Hospital of Copenhagen, Bispebjerg Bakke 23, DK-2400, Copenhagen, Denmark

[iv]Institute of Clinical Medicine, Faculty of Health, University of Copenhagen, Blegdamsvej 3B, DK-2200, Copenhagen, Denmark

*Correspondence to rasmus.rydbirk@regionh.dk

Supplementary Figures

Supplementary Figure 1

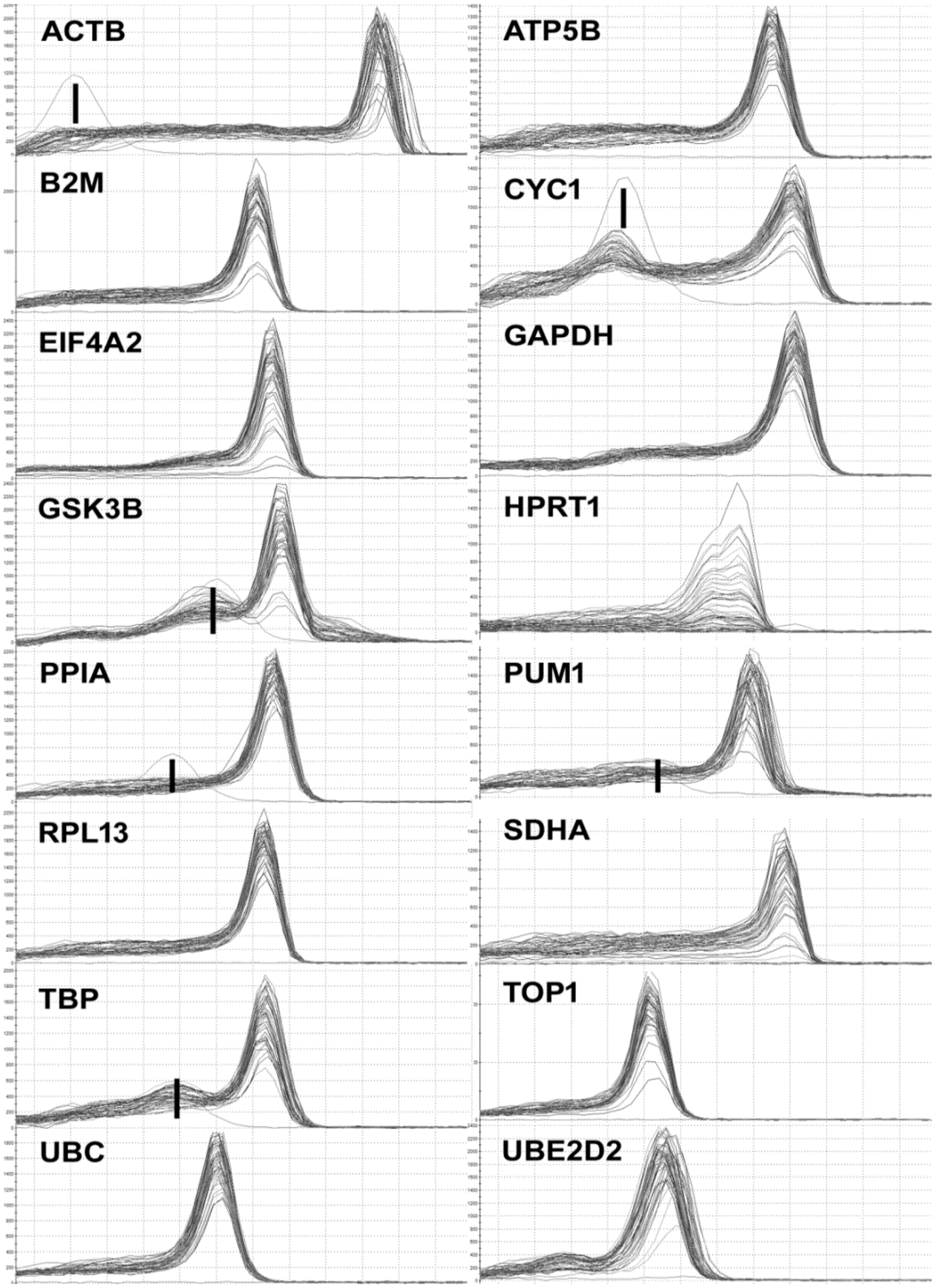

Figure S1: Melting curves for all analyzed candidate reference genes and the gene of interest, GSK3B. Black bars indicate the presence of primer dimers.

Supplementary Figure 2

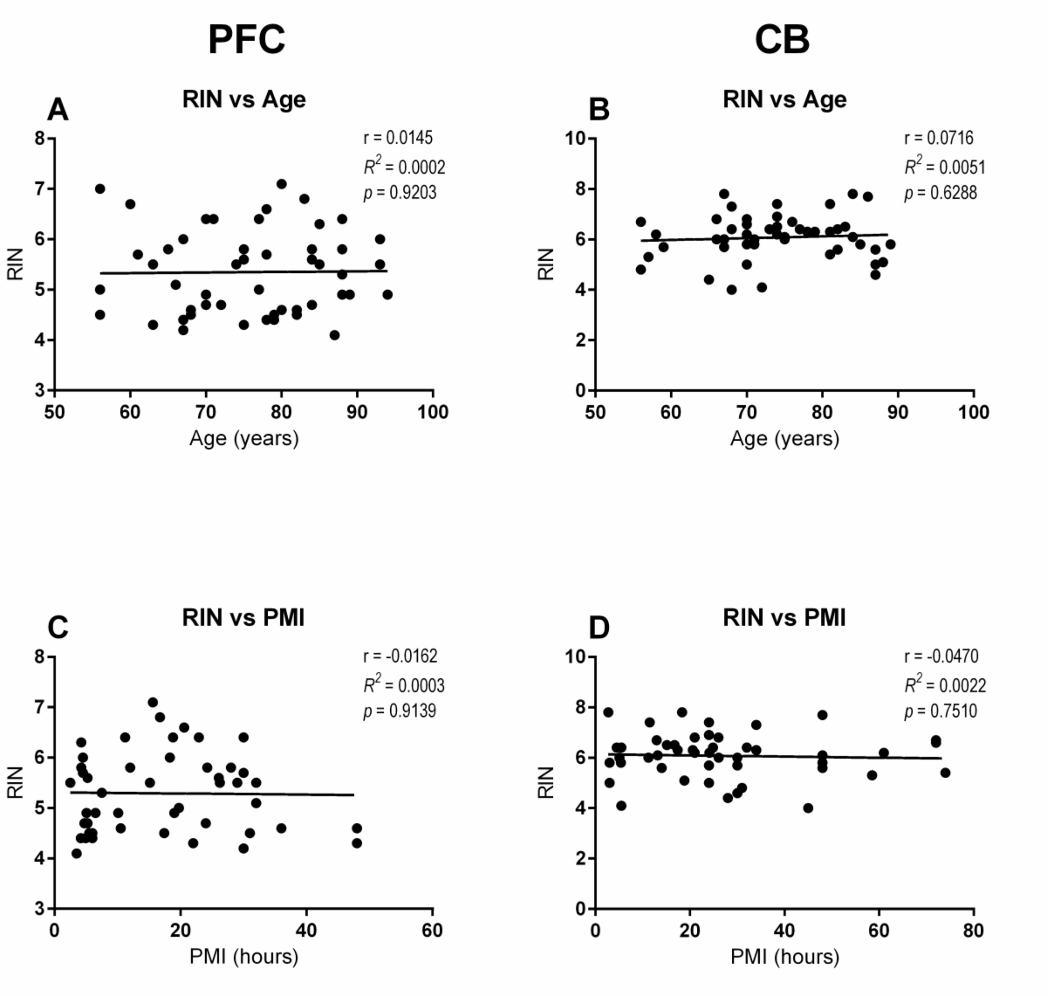

Figure S2: Correlations between RNA Integrity Number (RIN)-values, and age or post-mortem intervals (PMIs) in the prefrontal cortex (PFC), and the cerebellum (CB). Data were analyzed using Pearson product-moment correlation.

Supplementary Tables

Supplementary Table 1

Table S1: Demographic data for patient groups – the prefrontal cortex

Patient group	Origin	Sex	Age (years)	Braak stage	PMI (hours)	RIN
Normal, non-demented controls	NBB	F	68	1	5.5	4.5
	NBB	F	68	2	10.5	4.6
	NBB	M	70	2	10.1	4.9
	NBB	M	88	1	7.5	5.3
	NBB	F	93	1	4.5	6.0
	NBB	M	85	1	4.3	6.3
	NBB	F	84	1	4.8	4.7
	NBB	F	89	2	6.5	4.9
	NBB	F	77	1	19.8	5.0
	NBB	M	88	1	19.0	4.9
Alzheimer's disease	NBB	F	84	5	5.3	5.6
	NBB	F	78	5	4.5	5.7
	NBB	F	94	4	5.1	4.9
	NBB	M	78	5	6.0	4.4
	NBB	M	67	5	4.2	4.4
	NBB	M	87	6	3.5	4.1
	NBB	F	84	4	4.3	5.8
	NBB	M	72	5	5.3	4.7
	NBB	F	82	4	6.0	4.5
	NBB	F	93	4	2.5	5.5
Parkinson's disease	HV	F	80	-	15.6	7.1
	HV	M	74	-	15.2	5.5
	HV	F	85	-	26.3	5.5
	HV	M	88	-	18.8	6.4
	HV	F	79	-	5.0	4.4
	HV	M	88	-	24.3	5.8
	HV	M	79	-	17.4	4.5
	HV	M	77	-	11.2	6.4
	HV	M	75	-	26.1	5.6
	HV	M	70	-	22.9	6.4
Multiple System Atrophy	BBH	F	63	-	22.0	4.3
	BBH	M	60	-	96.0	6.7
	BBH	F	63	-	29.0	5.5
	BBH	F	61	-	30.0	5.7
	BBH	M	66	-	32.0	5.1
	BBH	M	74	-	32.0	5.5
	BBH	F	75	-	48.0	4.3
	BBH	M	56	-	72.0	5.0
	BBH	F	56	-	31.0	4.5
	BBH	F	70	-	24.0	4.7
Progressive supranuclear palsy	BBH	F	56	-	61.0	7.0
	BBH	M	65	-	28.0	5.8
	BBH	F	75	-	12.0	5.8
	BBH	M	82	-	48.0	4.6
	BBH	M	67	-	30.0	4.2
	BBH	M	71	-	30.0	6.4
	HV	M	78	-	20.6	6.6
	HV	M	83	-	16.8	6.8
	HV	M	67	-	18.3	6.0
	BBH	M	80	-	36.0	4.6

Sample origin: NBB: Netherlands Brain Bank, HV: Harvard Brain Bank, BBH: Bispebjerg-Frederiksberg Hospital Brain Bank; M: male; F: female; RIN: RNA integrity number; PMI: post-mortem interval.

Supplementary Table 2

Table S2: demographic data for patient groups – cerebellum

Patient group	Origin	Sex	Age (years)	Braak stage	PMI (hours)	RIN
Normal, non-demented controls	BBH	M	58	-	61.0	6.2
	BBH	F	70	-	72.0	6.6
	BBH	F	74	-	21.0	6.2
	BBH	F	74	-	24.0	7.4
	BBH	F	81	-	34.0	6.3
	BBH	F	70	-	21.0	6.8
	BBH	M	74	-	24.0	6.9
	BBH	M	86	-	48.0	7.7
	BBH	M	66	-	26.0	6.8
	BBH	F	68	-	32.0	6.4
Alzheimer's disease	NBB	F	77	5	4.5	6.4
	NBB	F	89	5	3.0	5.8
	NBB	F	85	6	3.0	5.8
	NBB	F	87	5	3.0	5.0
	NBB	F	84	5	2.8	7.8
	NBB	M	67	6	5.1	6.0
	NBB	M	73	4	5.5	6.4
	NBB	M	81	5	11.5	7.4
	NBB	M	72	4	5.5	4.1
	NBB	M	71	4	5.4	5.8
Parkinson's disease	HV	M	88	-	18.8	5.1
	HV	F	87	-	14.0	5.6
	HV	M	66	-	11.2	6.0
	HV	M	84	-	13.2	6.1
	HV	M	70	-	24.1	6.2
	HV	F	82	-	24.9	6.4
	HV	M	79	-	17.4	6.3
	HV	M	75	-	26.1	6.0
	HV	M	74	-	15.2	6.5
	HV	M	76	-	13.0	6.7
Multiple System Atrophy	BBH	F	75	-	-	6.1
	BBH	F	59	-	24.0	5.7
	BBH	M	56	-	72.0	6.7
	BBH	F	56	-	31.0	4.8
	BBH	F	70	-	48.0	5.8
	BBH	F	70	-	24.0	5.0
	BBH	M	57	-	58.5	5.3
	BBH	F	68	-	45.0	4.0
Progressive supranuclear palsy	BBH	M	65	-	28.0	4.4
	BBH	M	82	-	48.0	5.6
	BBH	F	87	-	30.0	4.6
	BBH	M	81	-	74.0	5.4
	BBH	M	67	-	30.0	5.7
	BBH	M	71	-	30.0	6.0
	BBH	F	68	-	34.0	7.3
	HV	M	78	-	20.6	6.3
	HV	M	83	-	16.8	6.5
	HV	M	67	-	18.3	7.8

Sample origin: NBB: Netherlands Brain Bank, HV: Harvard Brain Bank, BBH: Bispebjerg-Frederiksberg Hospital Brain Bank; M: male; F: female; RIN: RNA integrity number; PMI: post-mortem interval.

Supplementary Table 3

Group	RIN		Age (years)		PMI (hours)	
Prefrontal cortex	**Correlation**	***p*-value**	**Correlation**	***p*-value**	**Correlation**	***p*-value**
**NC (Raw Ct-values)**	-0.558	0.100*	-0.761	0.011	-0.199	0.583
**NC (Normalized)**	-0.261	0.467	0.338	0.339	0.234	0.515
**AD (Raw Ct-values)**	0.634	0.049	0.082	0.821	0.130	0.721
**AD (Normalized)**	-0.018	0.961	-0.624	0.054	-0.188	0.603
**PD (Raw Ct-values)**	-0.717	0.030	-0.471	0.200	0.078	0.842
**PD (Normalized)**	-0.085	0.834*	0.627	0.071	0.158	0.685
**MSA (Raw Ct-values)**	-0.343	0.367	0.273	0.478	0.012	0.990*
**MSA (Normalized)**	0.550	0.121	-0.077	0.845	0.326	0.390*
**PSP (Raw Ct-values)**	-0.737	0.015	0.378	0.282	-0.160	0.661
**PSP (Normalized)**	-0.113	0.755	-0.183	0.614	0.673	0.033
Cerebellum	**Correlation**	***p*-value**	**Correlation**	***p*-value**	**Correlation**	***p*-value**
**NC (Raw Ct-values)**	-0.254	0.474*	-0.375	0.286	-0.309	0.385
**NC (Normalized)**	0.096	0.792	-0.176	0.677	0.460	0.181
**AD (Raw Ct-values)**	-0.556	0.099*	-0.251	0.485	0.358	0.307*
**AD (Normalized)**	-0.768	0.010	-0.179	0.203	0.308	0.385*
**PD (Raw Ct-values)**	-0.344	0.331	0.223	0.535	0.376	0.284
**PD (Normalized)**	0.535	0.115*	-0.515	0.887	-0.172	0.635
**MSA (Raw Ct-values)**	-0.538	0.169	-0.075	0.860	-0.603	0.152
**MSA (Normalized)**	-0.059	0.890*	-0.193	0.582	0.507	0.246
**PSP (Raw Ct-values)**	0.036	0.933	0.382	0.351	0.073	0.887*
**PSP (Normalized)**	-0.731	0.039	0.450	0.033	0.561	0.163*

Table S3: Correlation analyses of the different patient groups and different experimental conditions. Correlations were performed for the RNA integrity number (RIN), age, and post-mortem intervals (PMIs) based on raw Ct-values from the reverse transcriptase quantitative real-time PCR and normalized to the most stable reference genes in the specific group. NC: Normal controls, AD: Alzheimer's disease; PD: Parkinson's disease; MSA: Multiple System Atrophy; PSP: Progressive Supranuclear Palsy. Bonferroni correction was used on Pearson product-moment or Spearman rank correlation (*) with *p*-values below 0.0025 considered significant.
